# Maboss for HPC environments: implementations of the continuous time Boolean model simulator for large CPU clusters and GPU accelerators

**DOI:** 10.1186/s12859-024-05815-5

**Published:** 2024-05-24

**Authors:** Adam Šmelko, Miroslav Kratochvíl, Emmanuel Barillot, Vincent Noël

**Affiliations:** 1 Department of Distributed and Dependable Systems, Charles University, Prague, Czech Republic; 2 Luxembourg Centre for Systems Biomedicine, University of Luxembourg, Esch-sur-Alzette, Luxembourg; 3 Institut Curie, Université PSL, 75005 Paris, France; 4 INSERM, U900, 75005 Paris, France; 5 Mines ParisTech, Université PSL, 75005 Paris, France

**Keywords:** Computational biology, High performance computing, Boolean models

## Abstract

**Background:**

Computational models in systems biology are becoming more important with the advancement of experimental techniques to query the mechanistic details responsible for leading to phenotypes of interest. In particular, Boolean models are well fit to describe the complexity of signaling networks while being simple enough to scale to a very large number of components. With the advance of Boolean model inference techniques, the field is transforming from an artisanal way of building models of moderate size to a more automatized one, leading to very large models. In this context, adapting the simulation software for such increases in complexity is crucial.

**Results:**

We present two new developments in the continuous time Boolean simulators: MaBoSS.MPI, a parallel implementation of MaBoSS which can exploit the computational power of very large CPU clusters, and MaBoSS.GPU, which can use GPU accelerators to perform these simulations.

**Conclusion:**

These implementations enable simulation and exploration of the behavior of very large models, thus becoming a valuable analysis tool for the systems biology community.

## Introduction

Biological systems are large and complex, and understanding their internal behavior remains critical for designing new therapies for complex diseases such as cancer. A crucial approach in this endeavor is building computational models from existing knowledge and analyzing them to find intervention points and to predict the efficacy of new treatments [1, 2]. Many different frameworks have been used to describe biological systems, from quantitative systems of differential equations to more qualitative approaches such as Boolean models [3]. While the former seems more adapted to represent complex behavior, such as non-linear dependencies, the latter is being increasingly used because of its capability to analyze very large systems. Many Boolean models have been built to describe biological systems to tackle a variety of problems: from understanding fundamental properties of cell cycle [4, 5] to advanced properties of cancer [6–8].

Historically, the task of building Boolean models involved reading an extensive amount of literature and summarizing it in a list of essential components and their interactions. More recently, database listings of such interactions [9, 10] and experimental information retrieval techniques on a bigger number of components were subjected to many advancements. Combined with the design of automatic methods for Boolean formulae inference from the constraints encoded in the knowledge and the experimental data [11–14], these new developments allows the construction of large Boolean models. While this effort faces many challenges, we believe it is a promising way to study the large-scale complexity of biological systems. However, in order to analyze the dynamic properties of such large Boolean models, we need to develop efficiently scalable simulation tools.

Here, we present adaptations of MaBoSS [15, 16]—a stochastic Boolean simulator that performs estimations of state probability trajectories based on Gillespie stochastic simulation algorithm [17]—to modern HPC computing architectures, which provide significant speedups of the computation, thus allowing scrutinization and analysis of much larger Boolean models. In particular, the problem of properly quantifying low abundant phenotypes [18] can now be tackled by making more realistic the large number of simulation needed to cover the space of possible trajectories. The main contributions comprise two new implementations of MaBoSS:

- MaBoSS.GPU, a GPU-accelerated implementation of MaBoSS, which is designed to exploit the computational power of massively parallel GPU hardware.
- MaBoSS.MPI, a parallel implementation of MaBoSS which can scale to multinode environments, such as large CPU clusters.

The source code of the proposed implementations is publicly available at their respective GitHub repositories.^1^ We also provide the scripts, presented plots, data and instructions to reproduce the benchmarks in the replication package.^2^

To showcase the utility of the new implementations, we performed benchmarking on both existing models and large-scale synthetic models. As the main results, MaBoSS.GPU provided over 200$\times$ speedup over the current version of MaBoSS on a wide range of models using contemporary GPU accelerators, and MaBoSS.MPI is capable of almost linear performance scaling with added HPC resources, allowing similar speedups by utilizing the current HPC infrastructures.

## Background

### Boolean signaling models

A Boolean signaling model consists of *n* *nodes*, which can represent a gene, protein or an event in a cell. Nodes are either active or inactive, gaining binary values 1 or 0 respectively. The *state* of the whole model is represented by a vector *S* of *n* binary values where $S_i$ represents the value of the *i*-th node. We denote the set of all possible states as ${\mathcal{S}} = \{0, 1\}^n$; thus $|{\mathcal{S}}| = 2^n$.

Interactions in the model are described as transitions between two states. A single state can have multiple transitions to other states with assigned transition probabilities. In turn, a Boolean network is represented as a directed weighted graph $G = ({\mathcal{S}}, \rho )$, where $\rho : {\mathcal{S}} \times {\mathcal{S}} \rightarrow [0, \infty )$ is a transition function generating *transition rates*. These rates define edge weights of *G*, which are used to compute the probability of a transition from state *S* to $S'$ in the following way:

1 $$\begin{aligned} P(S \rightarrow S') = \frac{\rho (S, S')}{\sum _{S'' \in {\mathcal{S}}} \rho (S, S'')}. \end{aligned}$$

For convenience, it holds that

2 $$\begin{aligned} \rho (S, S') = 0 \iff \text {there is no transition from}\ S\ \text {to}\ S'. \end{aligned}$$

### MaBoSS: Markovian Boolean stochastic simulator

MaBoSS simulates the *asynchronous update strategy*, where only a single node changes its value in each transition (as opposed to the *synchronous update strategy*, for which all nodes that can be updated are updated [15]). Therefore, there is a transition from *S* to $S'$ only if it holds that

3 $$\begin{aligned} \begin{aligned} S_j \ne S'_j&\text { for a given } j \\ S_i = S'_i&\text { for } i \ne j. \end{aligned} \end{aligned}$$

Consequently, *S* can have at most *n* possible transitions. In programming terms, $S'$ is obtained by flipping the *j*-th bit of *S*.

To determine the possible transition rates, each node follows the *Boolean logic* ${\mathcal{B}}_i: {\mathcal{S}} \rightarrow [0, \infty )$, which determines the expected Poisson-process rate of transitioning to the other value. If ${\mathcal{B}}_i(S) = 0$, then the transition at node *i* is not allowed in state *S*. Given this formalization, the simulation can be also viewed as a continuous-time Markov process.

The main computational part of the Boolean logic is its binary function $f: {\mathcal{S}} \rightarrow \{0, 1\}$, which consists of logical operators (such as *and*, *or*, *xor*, *not*) with nodes as operands. For example,

4 $$\begin{aligned} f_i(S) = (S_2 \wedge S_3) \vee S_4 \end{aligned}$$

is the binary function for node *i*, having nodes 2, 3 and 4 as its operands. The binary function of a node determines the value to which the node can transition. Thus, *S* can transition at node *i* at rate *r* only if $f_i(S) \ne S_i$. Concisely, ${\mathcal{B}}_i$ is defined as^3^

5 $$\begin{aligned} {\mathcal{B}}_i(S) = {\left\{ \begin{array}{ll} 0 &\quad{} \text {if } S_i = f_i(S) \\ r &\quad{} \text {otherwise} \end{array}\right. } \end{aligned}$$

MaBoSS algorithm simulates the above process to produce stochastic *trajectories*: sequences of states $S^0, S^1, \dots , S^k$ and time points $t^0< t^1< \dots < t^k$ where $t^0 = 0$ and $S^0$ is the initial state, and for each $i \in \{0, \dots , k-1\}$, $S^i$ transitions to $S^{i+1}$ at time $t^{i+1}$. The simulation ends either by a timeout when reaching the maximal allowed time, or by reaching a *fixed point* state with no outgoing transitions. The algorithm for a single iteration of the trajectory simulation is given explicitly in Algorithm 1, which is the direct application of the Gillespie stochastic simulation algorithm on the Boolean state space.

**Algorithm 1:**
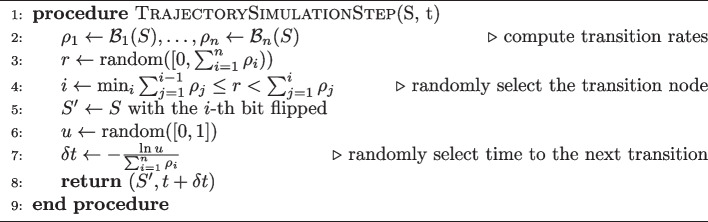
A single iteration of the MaBoSS simulation of a trajectory, given the state *S* and time *t*.

Multiple trajectories are generated and aggregated in compound trajectory statistics. Commonly obtained statistics include:

- *Network state probabilities on a time window*—Trajectory states are divided by their transition times into time windows based on the time intervals specified by a window size. For each window, the probability of each state is computed as the duration spent in the state divided by the window size. The probabilities of the corresponding windows are then averaged across all subtrajectories.
- *Final states*—The last sampled states from the trajectories are used to compute a final state distribution.
- *Fixed states*—All reached fixed points are used to compute a fixed state distribution.

To maintain the brevity in the statistics, MaBoSS additionally allows marking some nodes *internal*. This is useful because nodes that are not “interesting” from the point of final result view occur quite frequently in Boolean models, and removing them from statistics computation often saves a significant amount of resources.

### Computational complexity of parallel MaBoSS algorithm

#### Simulation complexity

We estimate the time required to simulate *c* trajectories as follows: For simplification, we assume that a typical Boolean logic formula in a model of *n* nodes can be evaluated in ${\mathcal{O}}(n)$ (this is a very optimistic but empirically valid estimate). With that, the computation of all possible transition rates (Algorithm 1, line 2) can be finished in ${\mathcal{O}}(n^2)$. The selection of the flipping bit (Algorithm 1, line 4) can be finished in ${\mathcal{O}}(n)$, and all other parts of the iteration can finish in ${\mathcal{O}}(1)$. In total, the time complexity of one iteration is ${\mathcal{O}}(n^2)$. If we simulate *c* trajectories with an upper bound of trajectory length *u*, the simulation time is in ${\mathcal{O}}(c \cdot u \cdot n^2)$.

In an idealized PRAM (parallel random access machine [19]) model with infinite parallelism, we can optimize the algorithm in the following ways:

- Given *c* processors, all trajectory simulations can be performed in parallel, reducing the time complexity to ${\mathcal{O}}(u \cdot n^2)$. (Note that this does not include the results aggregation. See *Statistics aggregation* section for further description.)
- With *n* processors, the computation of transition rates in the simulation can be done ${\mathcal{O}}(n)$ time, and the selection of the flipping bit can be done in ${\mathcal{O}}(\log {n})$ time using a parallel prefix sum, giving ${\mathcal{O}}(n)$ time for a single iteration.

Thus, using a perfect parallel machine with $c \cdot n$ processors, the computation time can be reduced to ${\mathcal{O}}(u \cdot n)$. Notably, the ${\mathcal{O}}(u)$ simulation steps that must be performed serially remain a major factor in the whole computation time.

#### Statistics aggregation

The aggregation of the statistics from the simulations is typically done by updating a shared associative structure indexed by model states, differing only in update frequency between the three kinds of collected statistics.

If the associative structure is implemented as a hashmap, the updates can be done in ${\mathcal{O}}(1)$ for a single process. With multiple processors, the algorithm may hold partial versions of the hashmap for each processor, and aggregate all of them at the end of the computation, which can be done in ${\mathcal{O}}(\log {c} \cdot m)$ using *c* processors, assuming the maximal size of statistic to be *m*.

As an interesting detail, the hash structures pose a surprising constant-factor overhead. In networks where most nodes are internal, the hash map may be replaced by a fixed-size multidimensional array that holds an element for all possible combinations of external node values (basically forming a multidimensional histogram). We discuss the impact of this optimization in *Implementation* section.

### MaBoSS CPU implementation

MaBoSS was initially developed as a single-core application, but swiftly, it was extended with a basic parallelism to exploit the multi-core nature of modern CPUs. In this parallel implementation, the simulation of trajectories and the statistics aggregation were distributed among multiple cores using POSIX threads. In the following sections of the papers, this implementation will serve as a baseline, and we will refer to it simply as the *CPU version*.

Each statistics data held by a thread is represented by a hash map with the keys as the states of the model and the values as a numerical value. Therefore, their aggregation from multiple trajectories of multiple threads is carried out by a well-researched parallel sum reduction. To better understand how a researcher can use MaBoSS output, in the following section, we discuss the differences between the statistics in greater detail and show the standard ways of their visualization.

#### Statistics output and visualization

**Fig. 1:**
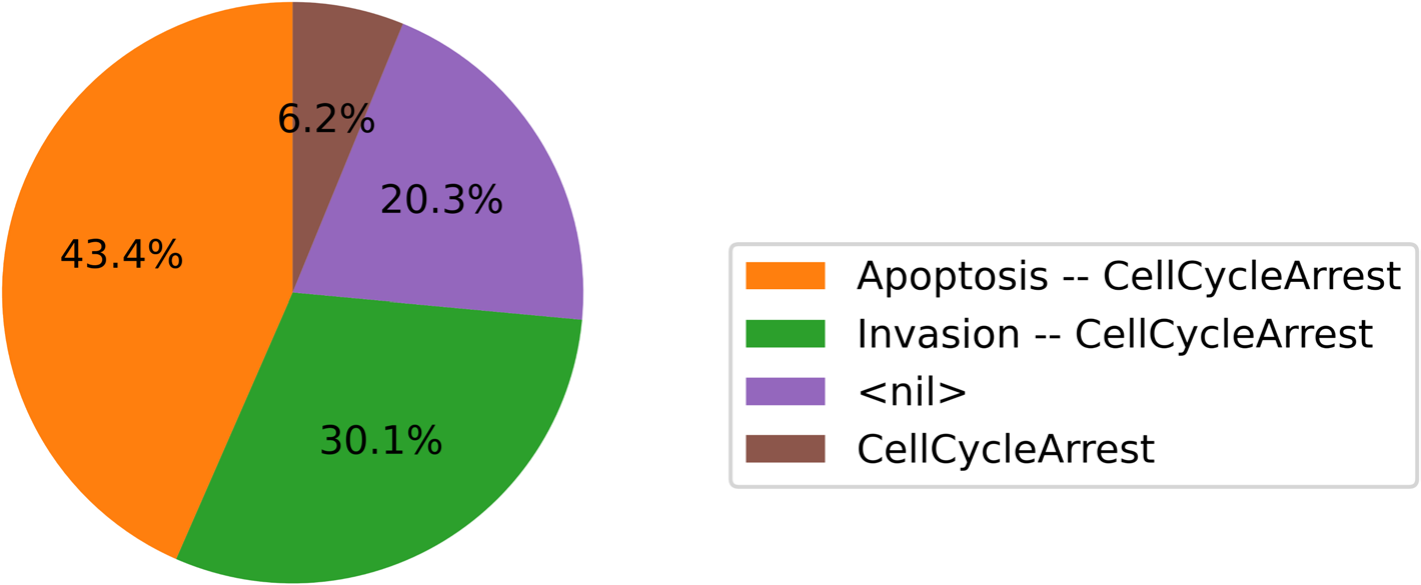
The final states pie chart shows the distribution of the last trajectory states. Labels denote which active non-internal nodes compose the state. *nil* label represents the state where all non-internal nodes are inactive

Each of the three kinds of statistics is in its nature a sample from a probabilistic distribution of Boolean states. This sample is represented in code as a hash map in the CPU version, varying in the (*key*, *value*) pairs according to the specific statistic. For the final states, the keys of the hash map are the model states, and the values are the number of times the state was sampled as the last in a trajectory. Such output can be visualized as a pie chart (see Fig. 1). The fixed states are represented similarly, but only the fixed points are stored in the hash map as keys.

**Fig. 2:**
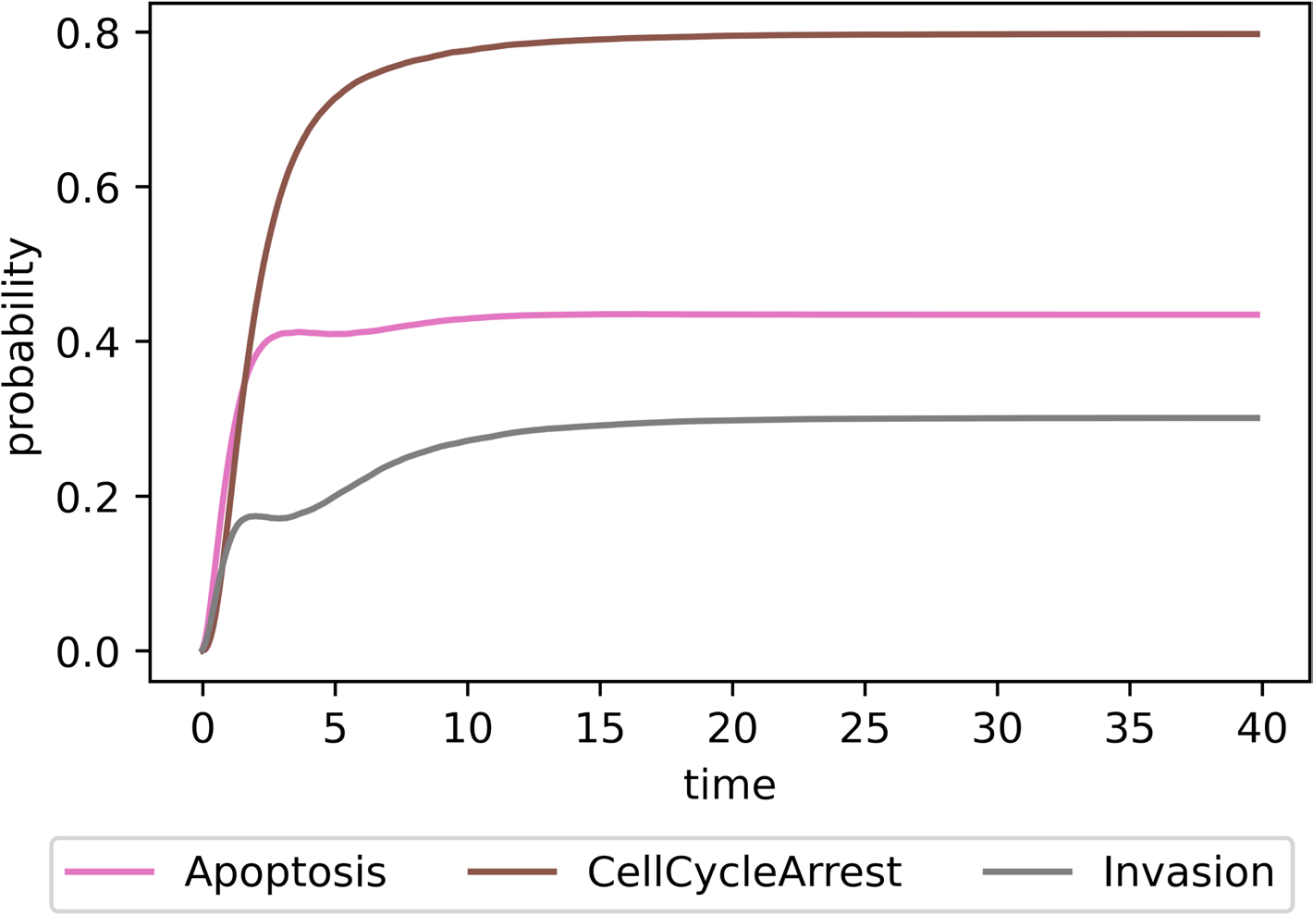
The line chart of trajectory state probabilities over time windows. Each line represents the ratio of an active non-internal node in the time window over all trajectories (e.g., at the beginning of the simulation, the *Apoptosis* node is inactive in all simulated trajectories and as the time reaches the value of 10, *Apoptosis* is active in around $40\%$ of trajectories). The x-axis represents the discrete simulation time with the window width of 0.1

The final and fixed state statistics characterize the behavior of the model at one point in time—at the end of the simulation. The network state probabilities on a time window highlight more dynamic characteristics of the model, showing how the average trajectory evolves over the simulation time. Programmatically, it is an extension of the final state statistics—instead of one hash map, there is a hash map for each time window. The hash map values are the state durations in the specific time window aggregated over all simulated trajectories. Further, these statistics can be visualized in various ways using a line chart. Figure 2 shows which non-internal nodes are active throughout the simulation.

As mentioned at the beginning of the section, if the trajectory does not reach a fixed point, the simulation is stopped after the maximal allowed time. This is a common scenario, especially when some trajectories form cycles, i.e., when a model has *cyclic attractor*, also known as limit cycles. A limit cycle is usually not directly visible from the state probability line charts; Stoll et al. [15] proposed methods to detect them (such as plotting the state and transition entropies), but we do not discuss the methods further in this paper for the sake of brevity.

## Implementation

### MaBoSS.GPU

#### Simulation

In the CPU version of MaBoSS, the simulation part is the most computationally demanding part, with up to 80% of MaBoSS runtime spent by just evaluating the Boolean formulae (the exact number depends on the model). The original formula evaluation algorithm in MaBoSS used a recursive traversal of the expression tree, which (apart from other issues) causes memory usage patterns unsuitable for GPUs: the memory required per each core is not achievable in current GPUs, and there are typically too many cache misses [20].

There are multiple ways to optimize the expression trees for GPUs: One may use a linked data structure that is more cache-friendly such as the van Emde Boas tree layout [21], or perhaps represent the Boolean formulae as a compact continuous array, or convert it to CNF or DNF (conjunctive or disjunctive normal form) bitmasks that can be easily evaluated by vector instructions. We decided to leave the exact representation choice on the compiler, by encoding the expressions as direct code and using the runtime compilation of GPU code [22]. In such an approach, the application reads the model files, writes the formulae as functions in CUDA C++ language, compiles them using the NVIDIA runtime compiler, and finally runs the simulation on GPU—all without user intervention.

Using this technique, the Boolean formulae are compiled as functions into a native binary code, which is directly executed by the GPU. As the main advantage, the formulae are encoded in the instructions, preventing unnecessary fetches of the encoded formulae from other memory. At the same time, the compiler may apply a vast spectrum of optimizations on the Boolean formulae, including case analysis and shortcutting, again resulting in faster evaluation.

A possible drawback of the runtime compilation stems from the relative slowness of the compiler—for small models, the total execution time of MaBoSS.GPU may be easily dominated by the compilation.

The work distribution was chosen to be one trajectory simulation per GPU thread. Due to the involved implementation complexity, we avoided optimization of the computation of individual trajectories by splitting the Boolean function evaluation into multiple threads (thus missing the factor of *n* threads from the asymptotic analysis). While such optimization might alleviate some cache pressure and thus provide significant performance improvements, we leave its exploration to future work.

#### Statistics aggregation

For optimizing the statistics aggregation, MaBoSS.GPU heavily relies on the fact that the typical number of non-internal nodes in a real-world MaBoSS model rarely exceeds 10 nodes, regardless of the size of the model. This relatively low number of states generated by non-internal nodes allows us to materialize the whole statistics structure (called “histogram”) as a fixed-size array (rarely exceeding $2^{10}$ elements).

This approach allows us to avoid storing the states as the keys and gives a simple approach that can map the state to the histogram index using simple bit masking and shifting instructions. Further, we use several well-known GPU histogram update optimizations to improve the performance, including shared memory privatization and atomic operations.

### MaBoSS.MPI

MaBoSS.MPI is a straightforward extension of the original MaBoSS CPU code to the MPI programming interface. Briefly, each MPI node is assigned to simulate the same number of trajectories (up to a remainder). These are further uniformly distributed among the CPU cores of the node, each thread progressively collecting the results into a privatized hashmap-based statistics aggregation structure.

Once all trajectory simulations are finished and the statistics are computed for each thread, the intermediate data are reduced into the final result using MPI collective operations.

## Results

To evaluate the impact of the implemented optimizations, we present the results of performance benchmarks for MaBoSS.GPU and MaBoSS.MPI by comparing their runtimes against the original CPU implementation. To obtain a comprehensive overview of achievable results, we used both real-world models and synthetic models with varying sizes.

### Benchmarking methodology

For the benchmarks, we used 3 real-world models of 10, 87 and 133 nodes (cellcycle [4], sizek [5] and Montagud [8]). In order to test the scalability of the GPU and MPI implementation, we also created several synthetic models with up to 1000 nodes. Synthetic models were designed in a way such that the length of each simulated trajectory is predictable, and the models have no stable states. The average length was arbitrarily set to 100, which creates reasonably-sized serial tasks to saturate the tested hardware well. Also, the number of non-internal nodes was kept low (5 nodes) to enable the usage of the histogram optimization. The synthetic models together with their Python generator are available in the replication package. Table 1 summarizes the main features of the benchmarked models.

The GPU implementation benchmarks were run on a datacenter-grade NVIDIA Tesla A100 GPU and a consumer-grade NVIDIA RTX 3070 Laptop GPU. The CPU implementation benchmarks were run on a 32-core Intel Xeon Gold 6130 CPU with multithreading. The CPU implementation was compiled with GCC 13.2.0, and the GPU implementation was compiled with CUDA 12.2. Each measurement was repeated 10 times, and the average runtime was used as the final result.

The MPI implementation benchmarks were run on the MareNostrum 4 supercomputer.^4^

**Table 1:** The main features of the synthetic and real-world models used in the benchmarks.

Model	# Nodes (non-inter.)	# Traj.	Avg. formula size	Avg. traj. length
cellcycle	10 (4)	1M	4	26
sizek	87 (4)	1M	22	525
Montagud	133 (3)	1M	4	197
*Synthetic*	10–1000 (5)	1–100M	10–100	100

It includes the size of models in terms of nodes and non-internal nodes, the number of simulated trajectories, the average formula size measured as the arithmetic mean of operands count in each formula, and the average length of all simulated trajectories. Note that not all combinations of features for the synthetic model were used in the benchmarks, see the following figures for more details

### Performance of MaBoSS.GPU

**Fig. 3:**
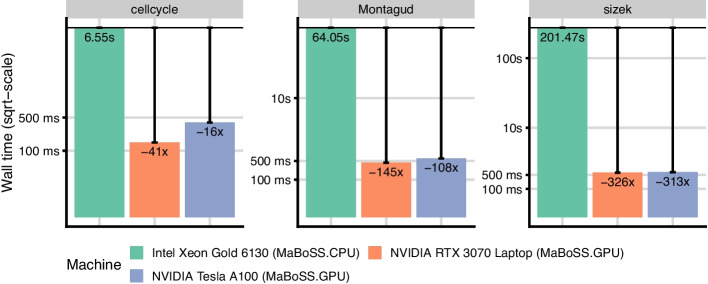
Wall time comparison of MaBoSS and MaBoSS.GPU on real-world models. Each model is simulated with 1 million trajectories

In Fig. 3, we compare the wall time of the CPU and GPU implementations on real-world datasets. The GPU implementation is faster than the CPU implementation on all models, and the speedup shows to be more significant on the models with more nodes and longer trajectories. On the Montagud model with 133 nodes, but a relatively short average trajectory, we achieve $145\times$ speedup. On a slightly smaller sizek model with a longer average trajectory, the speedup is up to $326\times$.

It is worth noting that the datacenter GPU performs worse than the laptop GPU. Both devices are bottlenecked by the runtime compilation of the Boolean formulae, however, NVIDIA A100 spends on average around 300ms more on the compilation step. Subtracting the compilation time, A100 is faster for all models. We did not spend time finding the root cause of this discrepancy since the value is negligible and the following benchmarks show that the runtime compilation overhead quickly disappears with increasing model size.

**Fig. 4:**
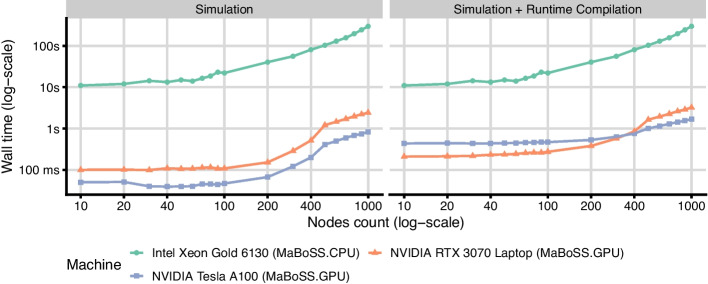
Wall time comparison of MaBoSS and MaBoSS.GPU on synthetic models with sizes ranging from 10 to 1000 nodes (x-axis) and the formula size of 10. Each model is simulated with 1 million trajectories. The two panels differ by the inclusion of the runtime compilation of the model logic, showing its impact on total run time

Figure 4 shows much finer performance progression on synthetic models. We observed that the CPU variant starts to progress steeper at around the 100 nodes boundary. We assume that the implementation hits the cache size limit, and the overhead of fetching the required data from the memory becomes dominant. The same can be observed in the GPU variant later at around 200 nodes. Expectably, the cache-spilling performance penalty is much more significant on GPUs. Overall, the results suggest that the optimization of dividing transition rate computations among multiple threads, as mentioned in *Implementation* section, may provide a better speedup for bigger models, as it alleviates the register and cache pressure.

**Fig. 5:**
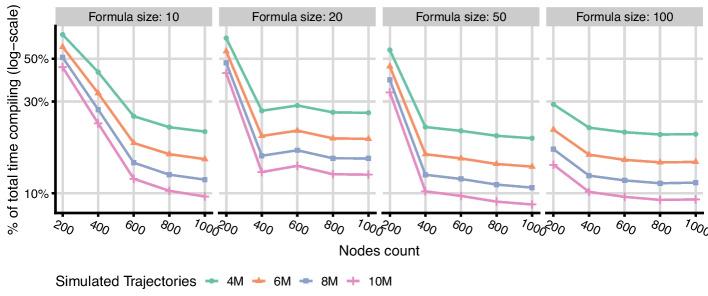
The ratio of time spent in the runtime compilation of the Boolean formulae in relation to the total runtime, simulating models with varying numbers of nodes, trajectories, and formula lengths

Additionally, Fig. 4 shows the total runtime of the GPU implementation including the runtime compilation step. Comparing the panels, we observe that the relative runtime compilation overhead quickly disappears with increasing model size. Figure 5 shows the results of more detailed benchmarks for this scenario, as run on the NVIDIA Tesla A100 GPU. We observed that the compilation time is linearly dependent on the number of nodes and formula lengths, which can be simply explained by the fact that these model properties extend source files that need to be compiled by a linear factor. Notably, as soon as the simulation becomes more computationally complex (e.g., by increasing the number of nodes, the number of simulated trajectories or their average length), the compilation time becomes relatively negligible even for models with unrealistically long formulae. This suggests that the runtime compilation is a viable optimization methodology also for much larger models.

### Performance of MaBoSS.MPI

**Fig. 6:**
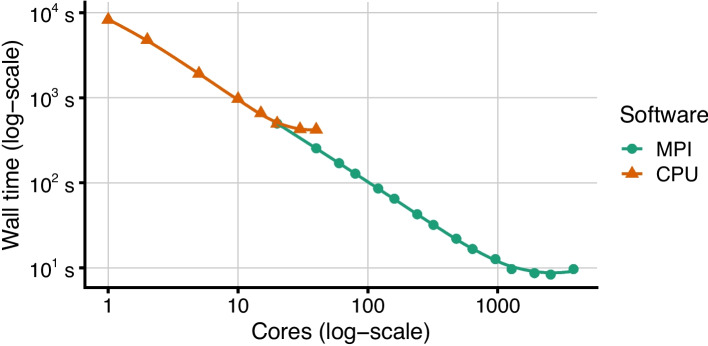
Scalability results of MPI implementation on Sizek model simulating 1 million trajectories with up to 192 MPI nodes and 20 cores per node, summing up to 3840 cores

Figure 6 shows the efficiency of the MaBoSS.MPI implementation on the sizek model. We ran multiple suites, ranging from a single MPI node up to 192 nodes, each running 20 cores. We can observe a close-to-linear speedup of up to 64 MPI nodes (1280 cores), and a plateau for larger suites (Fig. 6, green). This can be explained by hitting an expectable bottleneck in parallelization overhead and MPI communication cost when the problem is divided into too many small parts.

**Fig. 7:**
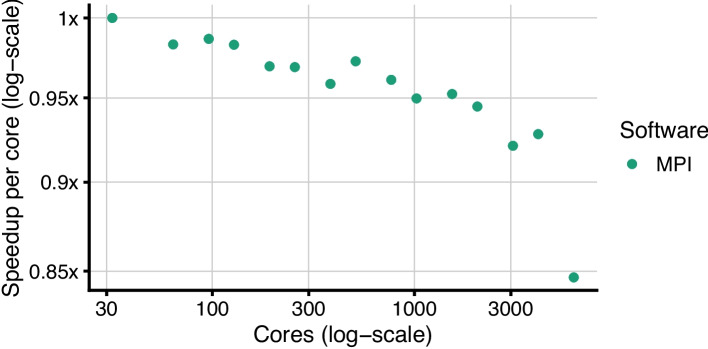
Speedup scaling of MPI implementation on the synthetic model with 1000 nodes, 100 million trajectories and the formula size of 10, running on up to 192 MPI nodes with 32 cores per MPI node, summing up to 6144 cores

To stress the scalability of the implementation, we also used the synthetic model with 1000 nodes running 100 million trajectories. We simulated this model on 32 cores per MPI node, on 1 to 192 nodes (32 to 6144 cores). The obtained speedups are summarized in Fig. 7. Using this configuration, the simulation time decreases from 20 h on 1 MPI node to 430 s on 192 nodes. As expected, the plateau in the speedup was observed only for much bigger suites. More specifically, we can see a pronounced decrease in the speedup at 192 nodes, hitting the aforementioned bottleneck during the utilization of more than 4096 cores.

## Conclusions

In this work, we presented two new implementations of MaBoSS tool, a continuous time Boolean model simulator, both of which are designed to enable utilization of the HPC computing resources: MaBoSS.GPU is designed to exploit the computational power of massively parallel GPU hardware, and MaBoSS.MPI enables MaBoSS to scale to many nodes of HPC clusters via the MPI framework. We evaluated the performance of these implementations on real-world and synthetic models and demonstrated that both variants are capable of providing significant speedups over the original CPU code. The GPU implementation shows 145–326$\times$ speedup on real-world models, and the MPI implementation delivers a close-to-linear strong scaling on big models.

Overall, we believe that the new MaBoSS implementations enable simulation and exploration of the behavior of very large, automatically generated models, thus becoming a valuable analysis tool for the systems biology community.

### Future work

During the development, we identified several optimization directions that could be taken by researchers to further scale up the MaBoSS simulation approach.

Mainly, the parallelization scheme used in MaBoSS.GPU could be enhanced to also parallelize over the evaluation of Boolean formulae. To avoid GPU thread divergence, this would however require a specialized Boolean formula representation, entirely different from the current version of MaBoSS; likely even denying the relative efficiency of the use of runtime compilation. On the other hand, this optimization might decrease the register pressure created by holding the state data, and thus increase the performance on models with thousands of nodes.

In the long term, easier optimization paths might lead to sufficiently good results: For example, backporting the GPU implementation improvements back to the MaBoSS CPU implementation could improve the performance even on systems where GPU accelerators are not available. Similarly, both MaBoSS.GPU and MaBoSS.MPI could be combined into a single software that executes distributed GPU-based analysis over multiple MPI nodes, giving a single high-performance solution for extremely large problems.

## Availability and requirements

- Project name: MaBoSS.GPU
- Project home page: https://github.com/sysbio-curie/MaBoSS.GPU
- Operating system(s): Platform independent
- Programming language: C++, CUDA
- Other requirements: Flex, Bison, CMake $>=$ 3.18, Cuda toolkit $>=$ 12.0
- License: MIT
- Any restrictions to use by non-academics: None
- Project name: MaBoSS.MPI
- Project home page: https://github.com/sysbio-curie/MaBoSS
- Operating system(s): Platform independent
- Programming language: C++
- Other requirements: Flex, Bison
- License: BSD3-clause
- Any restrictions to use by non-academics: None

## Data Availability

The scripts, presented plots, data and instructions to reproduce the benchmarks are available on GitHub [23].

## Abbreviations

HPC: High performance computing
CPU: Central processing unit
GPU: Graphical processing unit
MPI: Message passing interface
PRAM: Parallel random access machine
CNF: Conjunctive normal form
DNF: Disjunctive normal form

## Footnotes

1 https://github.com/sysbio-curie/MaBoSS.GPU, https://github.com/sysbio-curie/MaBoSS.

2 https://github.com/sysbio-curie/hpcmaboss-artifact.

3 Generally, ${\mathcal{B}}_i$ can be defined using a pair of binary formulas [15]: one used when a node is active and one when it is inactive. This results in a slightly more branched definition, which we omitted for brevity.

4 https://www.bsc.es/marenostrum/marenostrum
